# Usability evaluation of Alerta Alcohol 2.0: an eHealth game to prevent adolescent alcohol consumption

**DOI:** 10.1093/pubmed/fdag022

**Published:** 2026-03-24

**Authors:** Pablo Fernández-León, Marta Lima-Serrano, José Manuel Martínez-Montilla

**Affiliations:** Department of Nursing, Faculty of Nursing, Physiotherapy and Podiatry, University of Seville, Avenzoar St., 6, 41009, Sevilla, Spain; Red Cross University Nursing Centre, University of Seville, Cruz Roja Av., 1, 41009, Sevilla, Spain; Department of Nursing, Faculty of Nursing, Physiotherapy and Podiatry, University of Seville, Avenzoar St., 6, 41009, Sevilla, Spain; Institute of Biomedicine of Seville, IBiS/Virgen del Rocío University Hospital/CSIC, University of Seville, Campus Hospital Universitario Virgen del Rocío, Manuel Siurot St., s/n., 41013, Sevilla, Spain; Institute of Biomedicine of Seville, IBiS/Virgen del Rocío University Hospital/CSIC, University of Seville, Campus Hospital Universitario Virgen del Rocío, Manuel Siurot St., s/n., 41013, Sevilla, Spain; Virgen del Rocío University Hospital, Andalusian Health Service, Manuel Siurot St., s/n., 41013, Sevilla, Spain

**Keywords:** adolescents, alcohol use, binge drinking, computer tailoring, eHealth, usability testing

## Abstract

**Background:**

Perceived usability is a key determinant of eHealth intervention uptake. This study evaluated the usability of Alerta Alcohol 2.0, an animation-based, computer-tailored game designed to prevent alcohol use and binge drinking among adolescents.

**Methods:**

A cross-sectional mixed-methods approach was employed, combining a self-report questionnaire and cognitive debriefing using the think-aloud protocol.

**Results:**

Thirty-five participants (mean age = 16.57 years; 71.4% female) completed the five-session program. Results showed that older adolescents were more likely to recommend the intervention and perceived its length as more appropriate. Adolescents engaging in binge drinking responded more positively to the design and videos, found the difficulty level more suitable, and required less assistance. Participants with a positive general evaluation of the program scored higher on most of the 21 usability items than those with a negative evaluation. Key areas for improvement, such as repetitive questions, technical glitches, and text readability, were identified and addressed.

**Conclusions:**

This study highlights the value of a systematic feasibility process, rooted in evidence and informed by adolescents’ perspectives, to enhance program usability. Findings provide initial support for the usability of Alerta Alcohol 2.0 and inform future development of computer-tailored interventions targeting adolescent health behaviors.

## Introduction

Alcohol is the most commonly consumed psychoactive substance in Spain and a leading preventable risk factor for premature mortality and global disease burden.^1,2^ Problematic patterns such as binge drinking (BD) and high-intensity drinking (HID) carry long-term risks, including traffic accidents, violence, suicide, risky sexual behavior, academic failure, mental health disorders, and delinquency, as well as short-term effects like alcohol poisoning, loss of consciousness, and memory blackouts.^3,4^

Although adolescent alcohol use has declined in many Western countries, particularly among 12–17-year-olds,^5–7^ consumption among Spanish adolescents remains high, especially regarding BD.^1,7^ In the Spanish context, the standard drink contains 10 grams of ethanol, and the legal drinking age is 18; nevertheless, underage access to alcohol through nonauthorized means remains common.^8,9^ ESTUDES 2025 survey reported that 71.0% of adolescents aged 14–18 consumed alcohol in the past year, with 24.7% reporting BD in the last 30 days.^1^ Overall alcohol consumption is relatively balanced between genders, with males typically engaging in slightly higher levels of BD. Among Andalusian adolescents, HID prevalence was 1.3% in those aged 15–19, emphasizing the need for preventive interventions.^10^

Several digital approaches have been developed to address alcohol use, including web-based interventions and mobile health (mHealth) applications.^11^ However, most existing alcohol-related apps are commercial products designed primarily for adult users and tend to focus on self-monitoring, harm reduction, or treatment support rather than prevention among adolescents.^12,13^ In contrast, few rigorously evaluated eHealth tools are specifically tailored to young populations, particularly within the Spanish context, where school-based preventive interventions remain scarce.^10,14^

Alerta Alcohol 2.0 is a web-based computer-tailored (CT) intervention for school settings, aimed at preventing alcohol use and harmful patterns, including BD and HID, among Spanish adolescents. The program is implemented under the guidance of health professionals, ensuring that content and activities are appropriate and evidence-based. The platform is accessible across devices, although it is primarily delivered via computers given that using computers generally promotes greater task focus, which is particularly relevant when delivering a behavior-change intervention. Moreover, it aligns with common school policies restricting mobile phone use, minimizing potential safety concerns or implementation challenges, and taking advantage of school-provided computers to ensure feasible and standardized implementation. It incorporates animated videos and gamification strategies. It incorporates animated videos and gamification strategies^15^ and is a cultural adaptation of a Dutch intervention.^16,17^ Based on the Integrated Change (I-Change) model, the program evaluates participants’ behaviors and motivations, then delivers personalized advice and feedback through proprietary algorithms.^18^ CT interventions grounded in this model have shown (cost)effectiveness in modifying complex health behaviors, including alcohol consumption.^10,17,19^

Although CT interventions are increasingly popular and effective, participants may still encounter difficulties with interfaces, features, or content, leading to discontinuation.^20,21^ Usability studies, the most common form of user-based testing, evaluate whether users can access desired information in a prototype and guide iterative improvements.^22,23^ These studies identify challenges, assess engagement, and clarify user experiences, as perceived usability strongly affects both behavioral adoption and actual intervention use.^24,25^ Consequently, digital interventions should undergo beta testing in controlled real-world settings to refine content and examine factors influencing impact, such as participant characteristics.^26^ Various methods exist to assess user interface specifications,^27^ including think-aloud protocols^28^ and evaluations of usability factors in eHealth applications, typically categorized into ease of use, credibility, understandability, acceptability, and motivation.^29–31^

Putting all together, this study assessed the usability of Alerta Alcohol 2.0, an animation-based CT game designed to prevent alcohol use and BD among adolescents.

## Methods

### Study design

**Figure 1:**
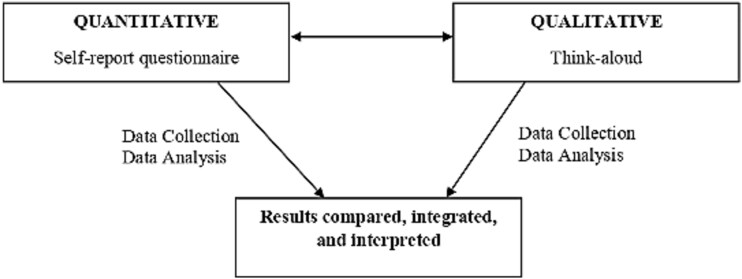
Triangulation of both methods with results.

A cross-sectional mixed-methods research (MMR) design was employed to evaluate usability among adolescents in October 2020. Participants completed a previously validated self-report questionnaire.^32^ Additionally, a cognitive debriefing approach was conducted using a think-aloud protocol^24^ to compare, integrate, and interpret the results (Fig. 1). The study adhered to MMR guidelines^33^ (Table S1).

All questionnaires and interview guides were originally developed in Spanish, based on the research team’s previous experience and on instruments used in earlier usability studies.^10,14,20,21,24,34,35^

### Participants and study procedures

Eligible participants were drawn from a larger sample of the main project, which ranged from 14 to 19 years old.^15^ For this usability study, we focused on participants aged 15–19 years, a range that aligns with the intervention’s target population and encompasses the period in which BD rises most sharply and reaches its highest prevalence before the end of secondary schooling.^1^ Usability studies typically involve small, focused samples; therefore, we selected one health-related vocational training group within this age range, part of the upper secondary level in the Spanish education system, which provided a relevant and practical context for testing the intervention.

Participants were recruited from one high school in Seville, Spain, with voluntary consent from students and parents. No financial incentives were provided, though participants benefited from Alerta Alcohol 2.0. A sample of 35 adolescents was selected, based on evidence that five participants suffice for usability testing and ~97% of issues are identified with 30 users.^36^ Recruitment was coordinated via school visits and phone contact by teachers and counselors.

Although the intervention was originally designed for delivery over six sessions on separate days,^37^ for this usability study, the first five sessions (excluding the follow-up evaluation questionnaire) were conducted across two school days. The intervention schedule was adapted for logistical reasons and to ensure consistent participation within the school timetable. Since the study aimed to assess usability rather than full intervention effects, a shorter administration period was considered appropriate. Sessions, each lasting ~1 hour, were conducted during regular class time with a teacher present, approved as part of school health education activities to avoid academic disruption (Fig. S1). Although Alerta Alcohol 2.0 includes both computer-based and mobile-adapted versions, the intervention in this study was administered exclusively through the computer-based format, taking advantage of the availability of school-provided computers. On Day 1, Session 1 (baseline questionnaire) and Session 2 (scenario 1) were administered, while Day 2 included Sessions 3 (scenarios 2 and 3), 4 (the challenge), and 5 (evaluation). All outcome measures were collected immediately after the fifth session to capture participants’ immediate usability experience.

Ethical approval was obtained from the Bioethical Committee of Andalusia (clinical trial registration number 2018I016). Data confidentiality and participant rights were ensured and explained to students and their parents or guardians, in compliance with EU Regulation 2016/679 and the Declaration of Helsinki. As the intervention focused on health education and prevention, risks were considered minimal. Teachers and researchers monitored participants’ comfort and engagement, and a referral protocol to school counselors and local health services was in place in case of distress or disclosure of problematic drinking. The intervention materials themselves included guidance and resources to support students. No adverse events occurred.

### Self-report questionnaire

#### Sociodemographic variables

included gender (male/female), age (years), educational level (e.g. first medium-grade vocational training: nursing assistant or pharmacy technician), and academic performance (from insufficient to outstanding).

#### Drinking behaviors

The study examined four distinct alcohol consumption patterns:

- *Any alcohol use:* “On which days during the past week did you consume alcohol?” (0 = no alcohol use; 1 = any alcohol use).
- *Weekly consumption*: number of alcoholic drinks reported for each day of the previous week. In accordance with Spanish public health guidelines,^8,9^ one standard alcoholic drink was defined as containing 10 g of pure ethanol. To facilitate participants’ understanding, visual guides and examples of common beverages (e.g. a 330 ml beer, a 140 ml glass of wine, or a 40 ml shot of spirits) were provided during questionnaire completion (Fig. S2).
- *BD* was evaluated using a gender-specific, open-ended item asking how many times in the past 30 days participants consumed four or more (girls) or five or more (boys) standard alcoholic drinks on a single occasion. Responses were categorized into a binary variable (0 = no BD; 1 = BD reported).
- *HID* was derived from daily alcohol intake reported for the previous week, classifying participants as engaging in HID if they consumed 8 or more standard drinks (girls) or 10 or more (boys) on a single occasion. This was dichotomized (0 = no HID; 1 = HID reported).

BD was assessed over the past 30 days, following the conventions of Spanish epidemiological studies,^10,14,35^ whereas HID was based on weekly data to capture short-term drinking patterns as reported in previous research.^38,39^

Although validated measures like the Timeline Follow-Back are common in adolescent alcohol research, this study used a brief, structured questionnaire suited to the school-based usability context. This approach enabled a concise assessment of recent drinking behaviors, consistent with previous studies in Spanish adolescents.^10,14,35^

#### Usability test

Usability testing was conducted using a 21-item questionnaire adapted from Saperstein *et al*.^32^ and previously tested in similar studies.^29,30^ It comprised three domains.

The ‘overall evaluation’ was measured using six items addressing visual design, avatars, animated videos, rewards, and narrative elements, rated on a five-point Likert scale (1 = Dislike a lot; 5 = Like a lot). An additional item evaluated the use of colloquial or youth-friendly language, rated from 1 (Totally disagree) to 5 (Totally agree).

The ‘overall perceived satisfaction’ was assessed using a single-item measure on a five-point Likert scale, ranging from 1 (Very dissatisfied) to 5 (Very satisfied).

The ‘program content’ was evaluated using five-point Likert scales (1 = Totally disagree; 5 = Totally agree) unless stated otherwise. Credibility was assessed with one item on content accuracy; understandability with two items on clarity; motivation with two items on willingness to reuse and recommend the program; and ease of use with two items on difficulty and need for assistance. Perceived impact was measured with four items on attitudes toward alcohol, perceived harm, self-efficacy in resisting peer pressure, and alcohol-related knowledge. Perceived interest was assessed via two items on advice usefulness and engagement, and acceptability with one item on program length (1 = Very long; 5 = Very short).

Higher scores on all items reflected more favorable evaluations of the program (i.e. better usability, acceptability, and satisfaction). No items required reverse scoring.

Internal consistency of the usability evaluation was high, with a Cronbach’s alpha of 0.853 overall, and reliability by domain ranged from 0.775 to 0.812, indicating good internal consistency across dimensions. A detailed description of all usability items, their response anchors, corresponding domains/subdomains, and Cronbach’s alpha coefficients for each domain is provided in Table S2.

### Think-aloud

The think-aloud study was conducted at the high school in presence of a researcher and a teacher. Adolescents were invited to log in, follow instructions, and complete Alerta Alcohol 2.0. Participants were encouraged to verbalize their thoughts and opinions to assess their reasoning and identify difficulties. To encourage open feedback, the researcher emphasized that the study’s objective was to evaluate the program, not participant behavior.

### Data analysis

In the quantitative phase, analyses were conducted using IBM SPSS Statistics 29 with no missing data. Descriptive statistics summarized participant characteristics, and associations between age, BD, and usability outcomes were analyzed using Student’s *t*-tests (*P* < .05). Because most participants were 16–17 years old and there were few cases at other ages, age was grouped into two ranges for descriptive purposes: 15–16 and 17–19. General evaluation scores were also dichotomized as negative vs. positive (median = 80) to facilitate straightforward comparisons between participants who rated the program more positively and those who rated it more negatively within this small usability sample. Differences in usability outcomes were assessed via Multivariate Analysis of Variance (MANOVA), followed by tests on individual variables.

In the qualitative phase, think-aloud feedback was transcribed, anonymized, and analyzed by a junior researcher, then reviewed by a senior researcher. A hybrid thematic analysis was conducted,^40^ predominantly inductive to allow unexpected usability issues to emerge, and subsequently organized within predefined usability domains. Feedback on content, including animated videos, was categorized into three main domains (overall evaluation, perceived satisfaction, program content) and their corresponding subdomains. Coding was conducted by one researcher and reviewed by a senior researcher to ensure reliability. Data saturation was reached when no new themes emerged.^41^ Participants’ insights were incorporated into Alerta Alcohol 2.0 refinements by team consensus.

Details on content and sources are provided in Tables S3 and S4.

## Results

### Self-report questionnaire

The study included a total of 35 adolescent participants, with 25 (71.4%) being female, and a mean age of 16.57 years (SD = 1.008). Regarding educational level, 21 (60%) were in the first medium-grade vocational training “Nursing assistant” and 14 (40%) were in “Pharmacy technician.” Concerning drinking behaviors, 25 (71.4%) reported any alcohol use, 22 (62.9%) reported BD in the last 30 days, and 3 (8.6%) reported HID. Table 1 describes the rest of the characteristics of the study sample.

**Table 1:** Characteristics of participants (sociodemographic variables and drinking behaviors).

Variables	Frequency (*N*)	Percentage (%)
**Gender**		
Male	10	28.6
Female	25	71.4
**Age (mean, SD)**	16.57 (1.008)	
15 years	5	14.3
16 years	11	31.4
17 years	15	42.9
18 years	2	5.7
19 years	2	5.7
**Type of educational level**		
First medium-grade vocational training “Nursing assistant”	21	60.0
First medium-grade vocational training “Pharmacy technician”	14	40.0
**Academic performance**		
Insufficient (0–4)	2	5.7
Sufficient (5)	21	60.0
Good (6)	10	28.6
Notable (7–8)	1	2.9
Outstanding (9–10)	1	2.9
**Any alcohol use**		
No	10	28.6
Yes	25	71.4
**Weekly consumption (mean, SD)**	3.54 (7.437)	
**Binge drinking**		
No	13	37.1
Yes	22	62.9
**Binge drinking occasions (mean, SD)**	2.00 (2.262)	
**High-intensity drinking**		
No	32	91.4
Yes	3	8.6

SD, standard deviation.

Associations between age, BD status, and the 21 usability items were analyzed (Table 2). Older adolescents (17–19) scored higher in motivation and acceptability, being more likely to recommend the program (*P* = .046) and consider its length appropriate (*P* = .008) than younger adolescents (15–16). Binge drinkers also scored higher on most overall evaluation and ease-of-use items, particularly appreciating the program design (*P* = .022), videos (*P* = .029), difficulty level (*P* = .029), and needing less assistance (*P* = .011) than nonbinge drinkers. No significant associations were found for other sociodemographic or drinking variables.

**Table 2:** Associations between usability test items and age and binge drinking.

Items	Youngest (15–16 years)	Oldest (17–19 years)	Statistic		Binge drinkers	Nonbinge drinkers	Statistic	
	*N* = 16 (45.7%)	*N* = 19 (54.3%)	Student’s *t*-test	*P*	*N* = 22 (62.9%)	*N* = 13 (37.1%)	Student’s *t*-test	*P*
	*Mean (SD)*	*Mean* *(SD)*			*Mean* *(SD)*	*Mean* *(SD)*		
**Overall evaluation** ^**a**^								
• Do you like the design of the program (images, text, sequences, phases)?	4.31 (0.793)	4.16 (0.765)	0.586	.562	4.45 (0.596)	3.85 (0.899)	2.413	.022^*^
• Do you like the design of the characters (avatars)?	4.56 (0.629)	4.26 (0.733)	1.282	.209	4.55 (0.510)	4.15 (0.899)	1.652	.108
• Do you like the different videos?	3.88 (0.806)	3.58 (0.769)	1.110	.275	3.95 (0.653)	3.31 (0.855)	2.353	.029^*^
• Do you like the different rewards (cards)?	3.88 (0.500)	4.11 (0.737)	−1.059	.297	4.14 (0.468)	3.77 (0.832)	1.460	.163
• Do you like the different stories presented?	3.81 (0.655)	4.00 (0.882)	−0.702	.487	4.09 (0.426)	3.62 (1.121)	1.468	.164
• Is the language used in the program appropriate for you?	4.25 (0.683)	4.42 (0.507)	−0.849	.402	4.27 (0.631)	4.46 (0.519)	−0.911	.369
**Overall perceived satisfaction** ^**a**^								
• What is the overall degree of satisfaction perceived with the program?	4.19 (0.544)	3.79 (0.855)	1.606	.118	3.86 (0.774)	4.15 (0.689)	−1.115	.273
**Content of the program**								
**Credibility** ^**a**^								
• Do you consider the content of the sessions credible?	4.13 (1.147)	4.21 (0.918)	−0.245	.808	4.41 (0.666)	3.77 (1.363)	1.868	.071
**Understandability** ^**a**^								
• Is the advice understandable?	4.31 (0.793)	4.42 (0.507)	−0.472	.641	4.36 (0.658)	4.38 (0.650)	−0.092	.928
• Is the information organized clearly?	4.38 (0.719)	4.42 (0.507)	−0.222	.826	4.36 (0.658)	4.46 (0.519)	−0.458	.650
**Motivation** ^**a**^								
• Would you use the program again?	3.38 (0.957)	3.53 (1.073)	−0.436	.666	3.45 (0.739)	3.46 (1.391)	−0.017	.987
• Would you recommend the program to someone?	3.38 (1.147)	4.11 (0.937)	−2.074	.046^*^	3.82 (0.853)	3.69 (1.437)	0.327	.746
**Ease of use** ^**a**^								
• What is the degree of difficulty of the program?	4.38 (0.806)	4.11 (0.809)	0.984	.332	4.45 (0.739)	3.85 (0.801)	2.283	.029^*^
• Have you needed help (from the teacher or researcher) to complete the sessions?	3.00 (1.461)	2.47 (1.429)	1.075	.290	3.18 (1.435)	1.92 (1.115)	2.710	.011^*^
**Perceived impact** ^**a**^								
• Have you changed your attitude towards alcohol consumption or binge drinking?	3.50 (1.033)	3.47 (1.172)	0.070	.945	3.50 (0.802)	3.46 (1.506)	0.085	.933
• Have you changed your perception of damage?	3.56 (1.031)	4.11 (0.937)	−1.631	.112	3.91 (0.750)	3.77 (1.363)	0.393	.697
• Have you improved skills to avoid binge drinking?	3.81 (0.981)	4.00 (0.943)	−0.575	.569	3.95 (0.653)	3.85 (1.345)	0.322	.750
• Have you improved your knowledge about alcohol consumption and binge drinking?	3.63 (1.147)	4.00 (1.155)	−0.960	.344	4.09 (0.750)	3.38 (1.557)	1.534	.145
**Perceived interest** ^**a**^								
• Do you consider the intervention to be useful?	3.75 (1.125)	4.21 (0.918)	−1.334	.191	4.09 (0.811)	3.85 (1.345)	0.674	.505
• Have you found the different messages/advice interesting?	3.81 (0.981)	4.11 (0.937)	−0.902	.374	3.86 (0.889)	4.15 (1.068)	−0.866	.393
**Acceptability** ^**a**^								
• What do you think about the length of the program?	1.25 (0.447)	2.26 (1.447)	−2.893	.008^**^	1.77 (1.270)	1.85 (1.144)	−0.171	.865

^*^ *P* < .05; ^**^P < .01. SD, standard deviation.

^a^Absolute range for each item, 1–5.

Associations between negative or positive general evaluations and the 21 usability items, adjusted for age and BD, were examined (Table S5). Multivariate analysis showed that general evaluation significantly influenced usability scores: adolescents with a positive evaluation scored higher on nearly all items, except for the final item in the acceptability subdomain (Pillai’s Trace = 0.967, *F* = 17.759, *P* < .001, η_p_^2^ = 0.967).

### Think-aloud

Think-aloud feedback indicated that adolescents generally found the 2-day intervention favorable, practical, and easy to understand, appreciating its interactivity, gamified videos, and concise content. Participants highlighted the program’s effectiveness in providing information on alcohol risks, promoting alcohol-free alternatives, and supporting action planning. Suggestions for improvement included simplifying text, clarifying navigation, and addressing video pop-up issues. Feedback also reflected differences based on prior alcohol experience, with abstinent adolescents perceiving less added value and binge drinkers engaging more with action planning. All insights were incorporated into the refinement of Alerta Alcohol 2.0 (Table 3).

**Table 3:** Participant comments from the think-aloud study categorized by usability (sub)-domains.

Domains	Subdomains	Citations
**Overall evaluation**		*The layout of the program is attractive and up to date.* *The videos and the gamification strategies (e.g., avatars, rewards, and stories) are creative and drive more enthusiasm.* *The language used is familiar and accessible.*
**Overall perceived satisfaction**		*I think the program has met my needs.* *I do not feel that this questionnaire takes me seriously; it frequently repeats the same content, which I find frustrating.* *Although the questionnaire includes repetitive questions, these pertain to different sides, prompting thoughtful reflection on one’s drinking behaviors.*
**Content of the program**	**Credibility**	*The animations clearly illustrated alcohol and highlighted the importance of reducing or avoiding its use.* *The animated material describes alcohol consumption and BD well and emphasizes the importance of reducing it.*
	**Understandability**	*The advice is clear, and I have no difficulty completing the questions.*
	**Motivation**	*I would definitely use the program again if it were available to me.*
	**Ease of use**	*I do not need anyone’s help to complete the different sessions; it is easy and simple.* *The use of pop-up windows can be a good alternative for not losing access to the program.* *When I clicked to view the video, I accidentally exited the program, and I didn’t know how to get back.*
	**Perceived impact**	*The videos with sensitive material (e.g., discomfort, blood, and vomits) did not have much of an impact on me.* *The program would certainly be interesting for alcohol drinkers or those who do not have enough alcohol knowledge.* *I am going to implement some action plans to reduce alcohol consumption.* *Based on the information I have just received, I understand that there is no reason to feel ashamed for choosing to drink non-alcoholic beverages.* *This is the first time I realize that when I go out with my friends, I binge drink.* *Playing a soccer game the day after my best friend’s birthday party was a good reason not to drink too much.*
	**Perceived interest**	*Short, clear and good supporting feedback.* *Informative and useful, it is participant-friendly.* *I already have a lot of alcohol knowledge, so the intervention might be less effective for me. However, it would definitely be valuable for binge drinkers or those with less alcohol knowledge than I have.*
	**Acceptability**	*The session 1 (the baseline questionnaire) is too long.*

## Discussion

### Main finding of this study

This study evaluated the usability and feasibility of Alerta Alcohol 2.0, an animation-based CT game for adolescents. Overall usability was rated positively, with high levels of satisfaction, willingness to reuse the program, and intention to recommend it. Older adolescents and participants reporting BD consistently rated the intervention more favorably across several usability domains, including design, videos, difficulty level, and autonomy of use. These findings indicate that Alerta Alcohol 2.0 is well accepted by its intended users and particularly engaging for subgroups at higher risk, supporting its suitability for further evaluation.

### What is already known on this topic

In line with previous studies, older adolescents tend to evaluate digital prevention interventions more positively than younger ones, likely reflecting greater cognitive and psychosocial maturity.^42,43^ Similarly, adolescents engaging in BD often report higher engagement with interactive and gamified digital tools, perceiving them as more appealing and better tailored to their needs.^44,45^

Consistent with prior usability research, participants who provided a positive overall evaluation also tended to score higher on specific usability components.^20,21,24,34^ These findings reinforce existing evidence that early usability testing is a critical step in the development of effective digital public health interventions.^26^ According to established criteria for evidence-based prevention programs,^46^ a randomized controlled trial is warranted to assess the efficacy and (cost)effectiveness of Alerta Alcohol 2.0, as well as its uptake and target subgroups,^24^ in line with current digital health implementation frameworks.^47^

### What this study adds

This study adds evidence on the feasibility of implementing a web-based, animation- and gamification-based cognitive training intervention within a real-school setting. The web format ensured compatibility with school information technology systems and equitable access during class time, where mobile phone use is often restricted. Teacher-supervised delivery facilitated structured implementation and consistent user feedback.^48^

Importantly, the study highlights the value of conducting systematic usability evaluations before testing intervention effectiveness. Early identification of design strengths (e.g. animated videos, gamification) and areas for refinement (e.g. perceived session length, minor technical issues) allowed improvements to be incorporated prior to outcome evaluation, in line with prior usability research^20,21,24,34^ and established recommendations for digital health intervention development.^26^

### Limitations of this study

Several limitations should be acknowledged. First, the intervention was delivered in a compressed 2-day format rather than the originally planned six sessions,^15,37^ which may have influenced perceptions of session length. Second, the small, nonprobabilistic sample, appropriate for usability testing,^36^ limited statistical power and generalizability. Similarly, the predominance of female participants reflects the selected vocational programs and does not mirror national drinking patterns.^1^

Usability testing was conducted only on the computer-based version; therefore, findings may not generalize to mobile delivery, which should be examined in future studies.^49^ Additionally, BD and HID were assessed using different recall periods (30 days vs. 1 week), which may slightly limit their direct comparability. Also, age and general evaluation scores were categorized to facilitate descriptive comparisons in this exploratory usability study, which may have obscured linear relationships.

Finally, qualitative analysis of think-aloud data was initially conducted by a single researcher, although interpretations were reviewed collaboratively to minimize bias. We also acknowledge that participating students spent ~5 hours in program activities, which may have represented a minor opportunity cost in terms of regular classroom time but provided valuable knowledge and practical skills for alcohol risk prevention.

## Conclusion

Usability testing of the Alerta Alcohol 2.0 program enabled refinements to the intervention based on feedback from adolescents, with teachers facilitating the sessions and supporting the usability evaluation. The evaluation offered multiple perspectives on the program’s strengths and areas for improvement, highlighting its feasibility and overall acceptability in a school setting. As such, this study provides preliminary support for the implementation of a web-based, computer-based intervention aimed at preventing alcohol use and BD among adolescents, and lays the groundwork for future, larger-scale evaluations to assess efficacy and (cost-)effectiveness.

## Supplementary Material

fdag022_Supplementary_material

## Data Availability

The raw data (e.g. results of self-report questionnaire or think-aloud sessions) that support the findings of this study are available on reasonable request from the corresponding author (M.L.-S.).
