# Supplementary material for: Usability evaluation of Alerta Alcohol 2.0: an eHealth game to prevent adolescent alcohol consumption
Source: J Public Health (Oxf). 2026 Mar 24;48(2):477–87. doi: 10.1093/pubmed/fdag022 (PMC13223592; doi:10.1093/pubmed/fdag022)
Supplement: fdag022_Supplementary_material [file fdag022_supplementary_material.zip › Table S2. Description of usability scale items and scoring details.docx]

**Table S2.** Description of usability scale items and scoring details.

| **Domains** | **Subdomains** | **Items** | **Response anchors** | **Cronbach´s alpha** |
| --- | --- | --- | --- | --- |
| Overall evaluation | N/A | N = 6   - Do you like the design of the program (images, text, sequences, phases)? - Do you like the design of the characters (avatars)? - Do you like the different videos? - Do you like the different rewards (cards)? - Do you like the different stories presented? | 1 = Dislike a lot  2 = Dislike  3 = Neither like nor dislike  4 = Like  5 = Like a lot | 0.775 |
|  |  | - Is the language used in the program appropriate for you? | 1 = Totally disagree  2 = Partially disagree  3 = Neither agree nor disagree  4 = Partially agree  5 = Totally agree |  |
| Overall perceived satisfaction | N/A | N = 1   - What is the overall degree of satisfaction perceived with the program? | 1 = Very dissatisfied  2 = Dissatisfied  3 = Neither satisfied nor dissatisfied  4 = Satisfied  5 = Very satisfied | N/A |
| Content of the program | Credibility | N = 14   - Do you consider the content of the sessions credible? | 1 = Totally disagree  2 = Partially disagree  3 = Neither agree nor disagree  4 = Partially agree  5 = Totally agree | 0.812 |
|  | Understandability | - Are the advices understandable? - Is the information organized clearly? | 1 = Totally disagree  2 = Partially disagree  3 = Neither agree nor disagree  4 = Partially agree  5 = Totally agree |  |
|  | Motivation | - Would you use the program again? - Would you recommend the program to someone? | 1 = Totally disagree  2 = Partially disagree  3 = Neither agree nor disagree  4 = Partially agree  5 = Totally agree |  |
|  | Ease of use | - What is the degree of difficulty of the program? - Have you needed help (from the teacher or researcher) to complete the sessions? | 1 = Very difficult  2 = Difficult  3 = Neither easy nor difficult  4 = Easy  5 = Very easy |  |
|  | Perceived impact | - Have you changed your attitude towards alcohol consumption or binge drinking? - Have you changed your perception of damage? - Have you improved skills for to avoid binge drinking? - Have you improved your knowledge about alcohol consumption and binge drinking? | 1 = Totally disagree  2 = Partially disagree  3 = Neither agree nor disagree  4 = Partially agree  5 = Totally agree |  |
|  | Perceived interest | - Do you consider the intervention to be useful? - Have you found the different messages/advices interesting? | 1 = Totally disagree  2 = Partially disagree  3 = Neither agree nor disagree  4 = Partially agree  5 = Totally agree |  |
|  | Acceptability | - What do you think about the length of the program? | 1 = Very long  2 = Long  3 = Neither short nor long  4 = Short  5 = Very short |  |

N/A: Not applicable

No reverse-coded items

All response anchors were rated on a five-point Likert scale

Cronbach´s alpha of 0.853 for overall usability scale
