# Supplementary material for: Usability evaluation of Alerta Alcohol 2.0: an eHealth game to prevent adolescent alcohol consumption
Source: J Public Health (Oxf). 2026 Mar 24;48(2):477–87. doi: 10.1093/pubmed/fdag022 (PMC13223592; doi:10.1093/pubmed/fdag022)
Supplement: fdag022_Supplementary_material [file fdag022_supplementary_material.zip › Table S3. Self-report questionnaire.docx]

**Table S3.** Self-report questionnaire.

| **Questions** | **Answers** | | **Variable type** |
| --- | --- | --- | --- |
| **Sociodemographic variables** | | | |
| Gender | 1 = Male; 2 = Female | | Nominal binary |
| Age | It was extracted from the period between the self-report date and the date of birth of the participant | | Quantitative |
| Type of educational level | 1 = Nursing assistant; 2 = Pharmacy technician | | Nominal binary |
| Academic performance | 1 = Insufficient (0 to 4); 2 = Sufficient (5); 3 = Good (6); 4= Notable (7-8); 5 = Outstanding (9-10) | | Ordinal |
| **Drinking behaviors** | | | |
| Any alcohol use | On which days during the past week did you consume alcohol?  1 = Monday; 2 = Tuesday; 3 = Wednesday; 4 = Thursday; 5 = Friday; 6 = Saturday; 7 = Sunday; 8 = I didn't drink alcohol last week; 9 = I never drink alcohol  Responses were categorized into a binary variable (0 = no alcohol use; 1 = any alcohol use). | | Nominal binary |
| How many standard alcoholic drinks did you consume last week? ^a^ | Answer with a number (e.g., 0, 1, 2, etc.)  Monday; Tuesday; Wednesday; Thursday; Friday; Saturday; Sunday | | Quantitative |
| Binge drinking | In the last 30 days, have you consumed 4 or more standard alcoholic drinks (if you are a girl) or 5 or more standard alcoholic drinks (if you are a boy) on a single occasion (for example at a party or at night)?  Responses were categorized into a binary variable (0 = no BD; 1 = BD reported). | | Nominal binary |
| Binge drinking occasions | In the last 30 days, how many times have you consumed 4 or more standard alcoholic drinks (if you are a girl) or 5 or more standard alcoholic drinks (if you are a boy) on a single occasion (for example at a party or at night)? Answer with a number (e.g., 0, 1, 2, etc.) | | Quantitative |
| **Usability test** | | | |
| Overall evaluation | • Do you like the design of the program (images, text, sequences, phases)?  • Do you like the design of the characters (avatars)?  • Do you like the different videos?  • Do you like the different rewards (cards)?  • Do you like the different stories presented? | 1 = Dislike a lot; 2 = Dislike; 3 = Neither like nor dislike; 4 = Like; 5 = Like a lot | Quantitative |
|  | • Is the language used in the program appropriate for you? | 1 = Totally disagree; 2 = Partially disagree; 3 = Neither agree nor disagree; 4 = Partially agree; 5 = Totally agree |  |
| Overall perceived satisfaction | • What is the overall degree of satisfaction perceived with the program? | 1 = Very dissatisfied; 2 = Dissatisfied; 3 = Neither satisfied nor dissatisfied; 4 = Satisfied; 5 = Very satisfied | Quantitative |
| Content of the program: Credibility | • Do you consider the content of the sessions credible? | 1 = Totally disagree; 2 = Partially disagree; 3 = Neither agree nor disagree; 4 = Partially agree; 5 = Totally agree | Quantitative |
| Content of the program: Understandability | • Are the advices understandable?  • Is the information organized clearly? | 1 = Totally disagree; 2 = Partially disagree; 3 = Neither agree nor disagree; 4 = Partially agree; 5 = Totally agree | Quantitative |
| Content of the program: Motivation | • Would you use the program again?  • Would you recommend the program to someone? | 1 = Totally disagree; 2 = Partially disagree; 3 = Neither agree nor disagree; 4 = Partially agree; 5 = Totally agree | Quantitative |
| Content of the program: Ease of use | • What is the degree of difficulty of the program?  • Have you needed help (from the teacher or researcher) to complete the sessions? | 1 = Very difficult; 2 = Difficult; 3 = Neither easy nor difficult; 4 = Easy; 5 = Very easy | Quantitative |
| Content of the program: Perceived impact | • Have you changed your attitude towards alcohol consumption or binge drinking?  • Have you changed your perception of damage?  • Have you improved skills for to avoid binge drinking?  • Have you improved your knowledge about alcohol consumption and binge drinking? | 1 = Totally disagree; 2 = Partially disagree; 3 = Neither agree nor disagree; 4 = Partially agree; 5 = Totally agree | Quantitative |
| Content of the program: Perceived interest | • Do you consider the intervention to be useful?  • Have you found the different messages/advices interesting? | 1 = Totally disagree; 2 = Partially disagree; 3 = Neither agree nor disagree; 4 = Partially agree; 5 = Totally agree | Quantitative |
| Content of the program: Acceptability | • What do you think about the length of the program? | 1 = Very long; 2 = Long; 3 = Neither short nor long; 4 = Short; 5 = Very short | Quantitative |

^a^ The “weekly consumption” and “high-intensity drinking” variables were created from this variable
