# Supplementary material for: Usability evaluation of Alerta Alcohol 2.0: an eHealth game to prevent adolescent alcohol consumption
Source: J Public Health (Oxf). 2026 Mar 24;48(2):477–87. doi: 10.1093/pubmed/fdag022 (PMC13223592; doi:10.1093/pubmed/fdag022)
Supplement: fdag022_Supplementary_material [file fdag022_supplementary_material.zip › Table S4. Think-aloud protocol.docx]

**Table S4.** Think-aloud protocol.

| **Core interview question** | **Probes/Follow-up questions (Prompts)** |
| --- | --- |
| *Overall evaluation* | |
| What is your overall perception of the design and presentation of the Alerta Alcohol 2.0 program? | - How do you assess the program’s visual design (layout)? - What are your thoughts on the gamification elements (avatars, rewards, stories)? - Do you find the language employed to be familiar and accessible? - Are there any sections that required modification of the language (e.g., shortening or rephrasing feedback messages)? |
| *Overall perceived satisfaction* | |
| Following the completion of the program, do you feel that it met your needs or expectations concerning information on alcohol consumption? | - Overall, how do you rate your experience? Is it favorable and practical? - What is your perspective on the necessity of content repetition? |
| *Content of the program: Credibility* | |
| How accurate and trustworthy do you perceive the content of the sessions and the information provided? | - Do you believe the animated material clearly illustrates alcohol consumption? - Do you believe it highlights the significance of avoiding or reducing alcohol use? |
| *Content of the program: Understandability* | |
| How do you rate the clarity and comprehensibility of the advice and explanations provided by the program? | - Do you experience any difficulty in understanding or completing the questions? - Is the supporting feedback you received clear and easy to assimilate? |
| *Content of the program: Motivation* | |
| Would you use the program again if given the opportunity? | - Would you recommend Alcohol Alerta 2.0 to your peers or friends? Please elaborate on your reasoning |
| *Content of the program: Ease of use* | |
| Is the process of navigating and completing the different program sessions straightforward? | - Do you require assistance from anyone to complete the sessions? - What is your experience regarding navigation clarity? - Do you encounter any technical challenges? - Is the implementation of pop-up windows a helpful alternative for maintaining access? |
| *Content of the program: Perceived impact* | |
| Do you believe the program had a demonstrable impact on your perspective regarding alcohol consumption or your future action plans? | - Is there a key insight that helps you recognize your own consumption patterns, such as binge drinking? - Do the videos containing sensitive material (e.g., discomfort, blood, or vomit) affect you significantly? - Does the program assist you in considering alternative options to alcoholic beverages? - How beneficial is the feature for scheduling action plans for the day following a potential drinking event? |
| *Content of the program: Perceived interest* | |
| Do you deem the content to be useful in assisting you to reduce your alcohol consumption or BD? | - Do you find the program informative and remain engaged throughout the sessions? - Do you agree that the program is more valuable for binge drinkers or for participants with less knowledge about alcohol? |
| *Content of the program: Acceptability* | |
| How do you perceive the overall length of the program or the individual sessions? | - Do you think any of the sessions are too long? - Does the duration of the program seem appropriate? |
