# Supplementary material for: Usability evaluation of Alerta Alcohol 2.0: an eHealth game to prevent adolescent alcohol consumption
Source: J Public Health (Oxf). 2026 Mar 24;48(2):477–87. doi: 10.1093/pubmed/fdag022 (PMC13223592; doi:10.1093/pubmed/fdag022)
Supplement: fdag022_Supplementary_material [file fdag022_supplementary_material.zip › Table S5. Associations between general evaluation (negative-positive) and usability test items.docx]

**Table S5.** Associations between general evaluation (negative/positive) and usability test items.

| **Items** | **Negative** | | **Positive** | | **Statistic** | | |
| --- | --- | --- | --- | --- | --- | --- | --- |
|  | **N = 17**  **(48.6%)** | | **N = 18**  **(51.4%)** | | ***F*** | ***p*** | ***η_p_²*** |
|  | *Mean* | *SD* | *Mean* | *SD* |  |  |  |
| **Overall evaluation ^a^** | | | | | | | |
| • Do you like the design of the program (images, text, sequences, phases)? | 4.12 | 0.781 | 4.33 | 0.767 | 0.485 | .491 | 0.015 |
| • Do you like the design of the characters (avatars)? | 4.12 | 0.781 | 4.67 | 0.485 | 6.339 | .017* | 0.170 |
| • Do you like the different videos? | 3.82 | 0.728 | 3.61 | 0.850 | 1.089 | .305 | 0.034 |
| • Do you like the different rewards (cards)? | 3.82 | 0.529 | 4.17 | 0.707 | 2.238 | .145 | 0.067 |
| • Do you like the different stories presented? | 3.71 | 0.849 | 4.11 | 0.676 | 2.065 | .161 | 0.062 |
| • Is the language used in the program appropriate for you? | 4.00 | 0.500 | 4.67 | 0.485 | 17.145 | .000*** | 0.356 |
| **Overall perceived satisfaction ^a^** | | | | | | | |
| • What is the overall degree of satisfaction perceived with the program? | 3.59 | 0.795 | 4.33 | 0.485 | 14.258 | .001** | 0.315 |
| **Content of the program** | | | | | | | |
| **Credibility ^a^** | | | | | | | |
| • Do you consider the content of the sessions credible? | 3.65 | 1.169 | 4.67 | 0.485 | 10.951 | .002** | 0.261 |
| **Understandability ^a^** | | | | | | | |
| • Are the advices understandable? | 4.06 | 0.659 | 4.67 | 0.485 | 9.284 | .005** | 0.230 |
| • Is the information organized clearly? | 4.12 | 0.600 | 4.67 | 0.485 | 8.789 | .006** | 0.221 |
| **Motivation ^a^** | | | | | | | |
| • Would you use the program again? | 3.00 | 1.000 | 3.89 | 0.832 | 7.763 | .009** | 0.200 |
| • Would you recommend the program to someone? | 3.18 | 1.074 | 4.33 | 0.767 | 14.357 | .001** | 0.317 |
| **Ease of use ^a^** | | | | | | | |
| • What is the degree of difficulty of the program? | 4.47 | 0.717 | 4.00 | 0.840 | 4.407 | .044* | 0.124 |
| • Have you needed help (from the teacher or researcher) to complete the sessions? | 2.53 | 1.007 | 2.89 | 1.779 | 0.374 | .546 | 0.012 |
| **Perceived impact ^a^** | | | | | | | |
| • Have you changed your attitude towards alcohol consumption or binge drinking? | 2.82 | 0.951 | 4.11 | 0.832 | 17.227 | .000*** | 0.357 |
| • Have you changed your perception of damage? | 3.35 | 0.996 | 4.33 | 0.767 | 10.592 | .003** | 0.255 |
| • Have you improved skills for to avoid binge drinking? | 3.59 | 1.004 | 4.22 | 0.808 | 3.903 | .057 | 0.112 |
| • Have you improved your knowledge about alcohol consumption and binge drinking? | 3.29 | 1.047 | 4.33 | 1.029 | 8.269 | .007** | 0.211 |
| **Perceived interest ^a^** | | | | | | | |
| • Do you consider the intervention to be useful? | 3.41 | 1.064 | 4.56 | 0.616 | 14.845 | .001** | 0.324 |
| • Have you found the different messages/advices interesting? | 3.59 | 1.004 | 4.33 | 0.767 | 6.458 | .016* | 0.172 |
| **Acceptability ^a^** | | | | | | | |
| • What do you think about the length of the program? | 2.29 | 1.532 | 1.33 | 0.485 | 8.170 | .008** | 0.209 |

SD: Standard deviation; F: MANOVA F-value; η_p_²: Partial eta squared

^a^ Absolute range for each item, 1–5

*p < .05; **p < .01; ***p < .001

Models were adjusted for participants' age and binge drinking condition
