# Supplementary material for: Usability evaluation of Alerta Alcohol 2.0: an eHealth game to prevent adolescent alcohol consumption
Source: J Public Health (Oxf). 2026 Mar 24;48(2):477–87. doi: 10.1093/pubmed/fdag022 (PMC13223592; doi:10.1093/pubmed/fdag022)
Supplement: fdag022_Supplementary_material [file fdag022_supplementary_material.zip › Table S1. MMR Checklist.docx]

| **Elements** | **Description** | **Page number** |
| --- | --- | --- |
| Rational and description of MMR design | - Provide a clear statement of the study purpose - Explicitly describe the MMR design in accordance with Creswell’s (2015) typology and use a diagram to illustrate the relationship and sequence of qualitative and quantitative research components - Justify why the MMR design is appropriate for meeting the study purpose | Pages 3-4 |
| Transparency in describing method details | - Describe the study population(s) and sample(s); e.g., who, what, how many) - Describe the sampling procedures (including inclusion and exclusion criteria, recruitment) - Describe qualitative data collection processes (how often data were collected, who collected the data, what kind of data collection instruments were used, how data were recorded—e.g., notes, transcripts) - Describe quantitative data collection processes (how often data were collected, who collected the data, what kind of data collection instruments were used measurements, validity/reliability) - Describe qualitative data analysis processes (coding, single or multiple coders, credibility) - Describe quantitative data analysis procedures (missing data and how they are handled, statistical tests used) | Pages 4-7 |
| Integration of qualitative and quantitative research components | - Interpret qualitative analysis results with appropriate quotes if necessary - Interpret quantitative analysis results in consideration of statistical significance, selection bias, and threats to validity - Compare qualitative and quantitative results - Address divergencies and inconsistencies between qualitative and quantitative results | Pages 7-9 |

**Table S1.** MMR Checklist.
