# Supplementary figures and images for: Usability evaluation of Alerta Alcohol 2.0: an eHealth game to prevent adolescent alcohol consumption
Source: J Public Health (Oxf). 2026 Mar 24;48(2):477–87. doi: 10.1093/pubmed/fdag022 (PMC13223592; doi:10.1093/pubmed/fdag022)

**
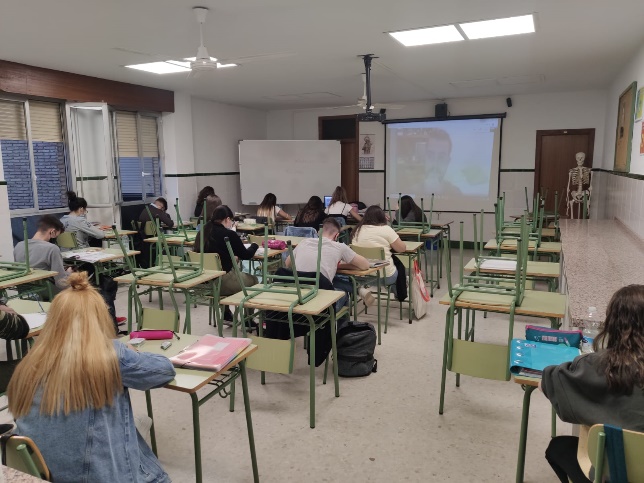
** **
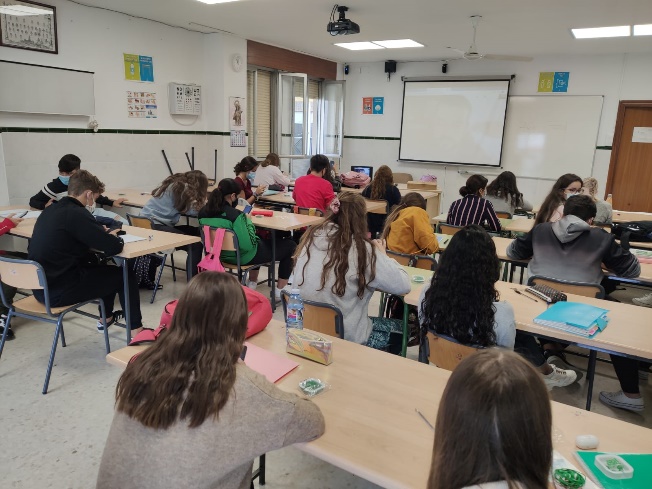
**

**Figure S1.** Classroom-based implementation of the Alerta Alcohol 2.0 usability study.

Supplement: fdag022_Supplementary_material [file fdag022_supplementary_material.zip › Figure S1. Classroom-based implementation of the Alerta Alcohol 2.0 usability study.docx]

**
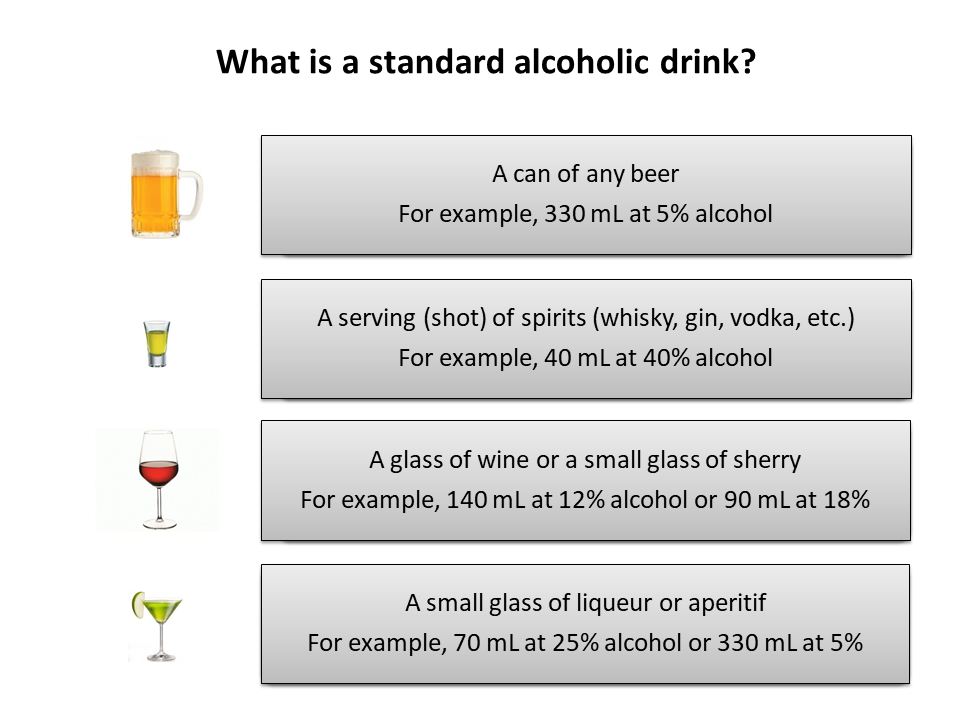
**

**Figure S2.** Examples of standard alcoholic drinks for participants.

Supplement: fdag022_Supplementary_material [file fdag022_supplementary_material.zip › Figure S2. Examples of standard alcoholic drinks for participants.docx]
